# Anemia in tuberculosis cases and household controls from Tanzania: Contribution of disease, coinfections, and the role of hepcidin

**DOI:** 10.1371/journal.pone.0195985

**Published:** 2018-04-20

**Authors:** Jerry Hella, Colin I. Cercamondi, Francis Mhimbira, Mohamed Sasamalo, Nicole Stoffel, Marcel Zwahlen, Thomas Bodmer, Sebastien Gagneux, Klaus Reither, Michael B. Zimmermann, Lorenz Risch, Lukas Fenner

**Affiliations:** 1 Ifakara Health Institute, Dar es Salaam, Tanzania; 2 Swiss Tropical and Public Health Institute, Basel, Switzerland; 3 University of Basel, Basel, Switzerland; 4 Laboratory of Human Nutrition, Institute of Food, Nutrition, and Health, ETH Zurich, Zurich, Switzerland; 5 Institute of Social and Preventive Medicine, University of Bern, Bern, Switzerland; 6 labormedizinisches zentrum Dr Risch, Liebefeld-Bern, Switzerland; 7 Gesundheitsamt, Kanton Solothurn, Switzerland; Medizinische Fakultat der RWTH Aachen, GERMANY

## Abstract

**Background:**

Tuberculosis (TB) induces a systemic inflammatory state affecting iron homeostasis. Patients with TB often have additional comorbidities such as anemia which can result in poorer treat outcomes. We studied the contribution of anemia and the role of the iron regulatory hormone hepcidin among TB patients and household contacts.

**Methods:**

We analyzed serum samples from 102 TB cases and 98 controls without TB, matched by age/sex, for hepcidin, iron, and inflammation parameters. Five controls developed TB within 12 months. We used linear regression to assess associations.

**Results:**

Anemia of chronic disease (ACD) was more frequent among cases than controls (59.8% vs. 26.1%), but iron-deficiency anemia more frequent in controls (10% vs. 1%). The median hepcidin level was higher in cases than controls (63.7 vs. 14.2 ng/mL), but coinfections with HIV, helminths, and respiratory pathogens did not show cumulative effects. Hepcidin was associated with more severe TB symptom scoring (coefficient 0.8, 95% confidence interval [CI] 0.5–1.2) and higher mycobacterial load (0.7, 95% CI 0.4–1.0). Hepcidin was higher in TB cases and controls who developed TB compared to controls without TB (p<0.001), even when restricting to HIV-negative study participants.

**Conclusions:**

ACD was the predominate etiology in TB patients suggesting limited benefit from iron supplementation. Increased hepcidin levels long before active disease, indicating altered iron metabolism, may be a marker for developing disease among TB-exposed individuals. Clinical management of anemia and nutrition interventions in TB patients need to be considered to improve the clinical course and outcomes.

## Introduction

One-quarter of the world's population is estimated to be infected with *Mycobacterium tuberculosis* [1]. Patients with TB often have additional comorbidities such as anemia which can result in poor treatment outcomes [2,3]. The global prevalence estimation of anemia was 33% in 2010, with higher estimates in sub-Saharan Africa [4]. Anemia is mainly caused by low dietary iron intake and low bioavailability, but chronic parasitic infection and inflammation also contribute to the burden of disease [5]. Anemia of chronic disease (ACD), is primarily found in patients with chronic immune activation such as TB and HIV-positive patients [6].

TB induces a chronic systemic inflammatory state, which triggers hepcidin synthesis from hepatocytes and predominately macrophages, influencing iron homeostasis [7–9]. As the key regulator of iron homeostasis [7,10], hepcidin drives the process of ACD by restricting the availability of iron for erythropoiesis [11,12]. The hormone does this by internalizing and degrading the iron exporter ferroportin in macrophages, hepatocytes and enterocytes, which results in sequestration of iron into the reticuloendothelial system and reduced dietary iron absorption [7,8,13]. The acquisition of iron from the host is a crucial prerequisite for the survival and replication of many pathogens, including *M*. *tuberculosis* [10,14]. Clinical studies showed that iron deficiency (ID) and anemia were associated with increased TB recurrence and mortality in HIV-positive TB patients [2,3]. In addition, hepcidin possibly plays a role in the immune response by inhibiting invasion of pathogens, having shown direct antimicrobial activity [15,16].

We studied the contribution of ACD and iron deficiency anemia (IDA) in TB patients in a high TB incidence country, Tanzania, where we compared TB patients to persons in contact households who were free of active disease. We also investigated the association of hepcidin levels with coinfections, disease severity, and progression from TB infection to disease.

## Methods

### Study setting

Study participants were part of an ongoing prospective cohort of bacteriologically confirmed pulmonary TB patients in Tanzania (TB-DAR) initiated in October 2013. The study site is located in the densely populated Temeke district of Dar es Salaam. With approximately 4.4 million people, Dar es Salaam is a TB hotspot region in Tanzania notifying 22% of all TB cases in Tanzania [17]. The details of the study setting have been described previously [18–22]. Participants included as TB cases were sputum smear-positive adult TB patients (>18 years), while those included as controls were exposed household contacts who did not have active TB.

### Selection of study participants

Among 359 TB patients recruited between 2014 and 2015, we randomly selected 103 TB cases, and 103 household controls exposed to a TB case but without active disease, matched according to age and sex. The final analysis included 200 participants, 102 cases and 98 controls, for whom serum samples and hemoglobin results were available.

### Study procedures and data collection

TB patients and controls were evaluated at the time of TB diagnosis (cases) or recruitment (controls), at six months (after completion of treatment for TB patients), and at 12 months. Clinical data were collected at each visit, and biological samples at the time of recruitment, as previously described [18–20,23]. Briefly, data were captured using the OpenDataKit application (www.opendatakit.org) on Android tablets, and data quality was monitored in real-time using the *odk_planner* tool [21]. Serum samples were taken at the time of TB diagnosis before starting TB treatment (cases) or at the time of recruitment (controls), and stored at -80° C. Xpert MTB/RIF (Cepheid, USA) was used to rule out TB in controls. Stool and urine samples were collected for diagnosis of helminths at the time of recruitment [18,23], as were nasopharyngeal swabs (Copan, USA) to detect respiratory pathogens. All study participants were screened for malaria using a rapid diagnostic test (Diagnostics Malaria P.f. MRDT, ICT Diagnostics, South Africa).

### Laboratory investigations

Full blood counts were done with a MS4 Vet hematology analyzer (Diamond Diagnostics, Massachusetts, USA). These blood tests were performed at the Temeke Regional Referral Hospital laboratory and inflammation parameters were obtained at the Labor Risch, Bern (Switzerland) using the Siemens Nephelometer BN II (soluble transferrin receptor) and the Cobas 6000, Roche diagnostics, Switzerland (all other parameters). We used a commercial assay (DRG Hepcidin 25 bioactive, HS ELISA GmbH, Marburg, Germany) to determine hepcidin levels [24]. HIV screening was done using Alere Determine HIV rapid test, and the Uni-gold HIV (Trinity Biotech, USA) rapid test served as a confirmatory test in case of a positive screening test.

The Kato-Katz method (in triplicates), Baermann technique (in duplicates), urine filtration (in duplicates), and circulating cathodic rapid antigen test (POC-CCA; Rapid Medical Diagnostics, South Africa) were used to diagnose helminths (*Strongyloides stercoralis*, *Trichuris trichiura*, *Schistosoma mansoni*, *S*. *haematobium*, *Ascaris lumbricoides*, hookworm) [18,23]. We analyzed the nasopharyngeal swabs to detect respiratory pathogens using a multiplex real-time PCR with a broad panel of 16 viral (Anyplex II RV16) and seven bacterial (Allplex panel 4) respiratory pathogens (S1 Table) according to the manufacturer’s instructions (Seegene, Seoul, South Korea) [25].

### Definitions

World Health Organization (WHO) criteria were used to classify the severity of anemia: no anemia (hemoglobin [Hb] 13.0 g/dL for men, 12.0 g/dL for women), mild anemia (11.0–12.9 g/dL for men, 11.0–11.9 g/dL for women), moderate anemia (8.0–10.9 g/dL for men and women) or severe anemia (8.0 g/dL for men and women). We used published definitions of ACD and IDA [6], where, patients with anemia were then classified into one of three mutually exclusive groups [6,26]: ACD, IDA, or combined ACD and IDA (ACD+IDA) (S1 Fig). Briefly, ferritin levels were used to distinguish ACD from IDA: ferritin >336.2 ng/mL defined patients with anemia as ACD, and ferritin <30 ng/mL as IDA [6]. Hepcidin levels further distinguished between ACD and ACD+IDA. We also used IDA definitions based on single laboratory parameters [27–29].

The mycobacterial load in TB patients was defined based on sputum smear microscopy results (quantitative scoring) [30]. In order to grade the clinical severity of TB, we adopted a previously published clinical TB score [18,31], the score was then categorized into mild (score of 1–5) and severe (score of ≥6). Helminth infection was defined as infection with any helminth species, and respiratory infection as detection of any respiratory viral or bacterial pathogen.

### Statistical analysis

Cases and controls were compared using conditional regression which accounts for the matched study design. Other groups were compared using chi-square or Fisher’s exact (binary variables) and Kruskal-Wallis (continuous variables) tests. We also used nonparametric tests for trend analysis across ordered groups. In addition, we performed logistic regression models to determine associations between binary outcomes (anemia versus no anemia, ACD versus no anemia, anemia based on hepcidin levels versus no anemia) and patient characteristics (body mass index [BMI], viral respiratory and helminth infection), and conditional regression models for the outcome TB (case-control matching). Odds ratios (ORs) were presented as unadjusted ORs and adjusted for HIV and BMI (aOR). Finally, we performed linear regression to assess associations between log laboratory parameter (hepcidin, procalcitonin, and hemoglobin levels) and progression to disease (cases, controls with and without TB) and TB disease severity (symptom scoring, mycobacterial load), as well as to assess associations between log hepcidin levels and hematological parameters. All analyses were performed in Stata version 14.0 (Stata corporation, Texas, USA).

### Ethics approval

The study was approved by the institutional review board of the Ifakara Health Institute (IHI, reference no. IHI/IRB/04-2015), the Medical Research Coordinating Committee of the National Institute for Medical Research in Tanzania (NIMR, reference no. NIMR/HQ/R.8c/Vol. I/357), and the Ethics Committee of the Canton of Basel (EKNZ, reference no. UBE-15/42). All participants gave written informed consent before enrolment.

## Results

We analyzed data from 102 cases and 98 controls. The median age was 32.9 years (interquartile range [IQR] 26.2–40.1); 152 (76.0%) were male. Of the 24 HIV-positive participants (12.0%), two were on antiretroviral therapy at the time of recruitment. The median BMI was lower among cases than controls (17.4 vs. 25.2 kg/m2). Coinfections with HIV, helminths, and respiratory pathogens were equally distributed in cases and controls (Table 1). Malaria screening was negative in all study participants. Among the controls, 5 (5.1%) developed active TB during the follow-up time of 12 months, with a median time of seven months (range 5.5–8.0) between recruitment and TB diagnosis.

**Table 1:** Patient characteristics of tuberculosis (TB) patients (cases) and household contact controls without TB (controls) in Tanzania.

Characteristic	Cases	Controls	*P* value ^1^
**All**	**102**	**98**	
**Age**, years, median (IQR)	33.0 (26.0–40.0)	32.7 (26.2–40.1)	-
**Male sex**, n (%)	78 (76.5)	74 (75.5)	-
**BMI**, kg/m ^2^, median (IQR)	17.4 (15.8–19.2)	25.2 (22.1–28.5)	0.002
**Education**, n (%)			0.30
No/primary	78 (76.5)	80 (81.6)	
Secondary/university	24 (23.5)	18 (18.4)	
**Coinfections**, n (%)			
HIV infection	13 (12.8)	11 (11.2)	0.64
Helminth infection ^3^	32 (31.4)	21 (21.4)	0.14
Any viral respiratory pathogen ^4^	25 (24.5)	19 (19.4)	0.24
Any bacterial respiratory pathogen ^4^	41/98 (41.8)	18/29 (62.1)	0.59
Malaria	0	0	-

BMI, body mass index; IQR, interquartile range

^1^ Accounts for case-control matching (values for the matching variables age and sex are not shown)

^2^ At the time of tuberculosis diagnosis (cases) or enrolment (controls)

^3^ Soil-transmitted and intestinal helminths, as determined by stool microscopy, Baermann test, urine filtration, and rapid urine antigen test

^4^ As determined by a panel of 16 viral respiratory and seven bacterial species (molecular detection in nasopharyngeal specimens)

### Prevalence of anemia and hematological characteristics

The overall median Hb concentration was 12.5 g/dL (IQR 10.9–13.7), and significantly lower in cases compared to controls (12.05 vs. 13.0, p<0.001, Table 2). Prevalence of anemia was significantly higher in cases compared to controls (62.2% vs. 37.8%, p<0.001) (S2 Fig). Particularly moderate and severe anemia were more common in cases than controls.

**Table 2:** Hematological, iron, and inflammatory parameters among cases and controls.

Parameter	No. included ^1^	Median (IQR)		*P* value ^2^
(unit)	Cases / controls	Cases	Control	
Iron (μmol/L)	99 / 91	4.4 (3.3–7.1)	12.9 (8.9–17.3)	<0.001
Ferritin (ng/mL)	101 / 89	309.8 (162.2–601.2)	103.5 (59.5–159.5)	<0.001
sTfR (mg/L)	89 / 75	1.8 (1.4–2.2)	1.4 (1.2–1.8)	0.001
Transferrin (g/L)	99 / 94	1.6 (1.4–2.0)	2.5 (2.3–2.8)	<0.001
Hepcidin (ng/mL)	81 / 65	63.7 (22.0–121.9)	14.2 (4.5–27.4)	0.003
CRP (mg/L)	99 / 94	67.8 (36.5–116.9)	1.6 (0.6–6.0)	<0.001
Procalcitonin (μg/L)	90 / 68	0.07 (0.04–0.17)	0.019 (0.019–0.03)	0.009
Hemoglobin (g/dL)	102 / 98	12.05 (10.3–12.9)	13.0 (11.5–14.3)	<0.001
MCV (f/L)	102 / 98	75.5 (68.6–82.8)	81.2 (76.0–86.3)	0.001
MCH (pg/cell)	102 / 98	25.0 (22.4–27.5)	26.3 (23.8–29.1)	0.027
MCHC (g/dL)	102 / 98	33.1 (32.0–34.0)	32.7 (31.2–33.9)	0.014
Red blood cell distribution width (f/L)	63 / 82	14.9 (13.8–16.7)	14.7 (13.5–15.7)	0.064

CRP, C-reactive protein; MCV, mean corpuscular volume; MCH, mean corpuscular hemoglobin; MCHC, mean corpuscular hemoglobin concentration; sTfR, soluble transferrin receptor

^1^ Patients with an available laboratory result

^2^ Accounts for case-control matching

Hematological, iron, and inflammatory parameters are shown for TB cases and controls in Table 2 (stratified by sex in S3A Fig and S3 Table and by anemia severity in S2 Table and S3B Fig). The median soluble transferrin receptor (sTfR) concentrations were significantly higher among cases than controls (1.8 mg/L, IQR [1.4–2.2] vs. 1.4 mg/L, IQR [1.2–1.8], p = 0.001). sTfR concentrations were also higher in cases and controls who had a more severe degree of anemia (mild to severe) as opposed to study participants without anemia (S2 Table). Similarly, the levels of the acute phase proteins procalcitonin and C-reactive protein (CRP) were higher in cases than in controls (Table 2). Median CRP concentration was significantly lower among controls without anemia than it was among controls with mild and moderate/severe anemia (respectively 0.9 vs. 3.3 vs. 4.1 mg/L, p = 0.024). Among TB cases, CRP levels were elevated across groups (varying with anemia status), thus reflecting the stronger inflammatory response in TB patients induced by *M*. *tuberculosis* infection (S2 Table).

### Types of anemia and risk factors

ACD was the most common cause-specific type of anemia among study participants (81/185, 43.8%), and significantly more common among cases than controls (59.8% vs. 26.1%, overall p<0.001) as shown in Table 3. Multifactorial anemia (mixed ACD and IDA) was the second most common type of anemia, where 13 (7.0%) participants had this type of anemia. IDA anemia was the third most common type of anemia (10/185, 5.4%), while controls had a significantly higher proportion of IDA than cases (10.2% vs. 1%).

**Table 3:** Etiology of anemia and iron deficiency based on single laboratory parameters among TB cases and controls.

Classification	No. included ^1^	n (%)		*P* value ^2^
	Cases / Controls	Cases	Controls	
**Cause-specific anemia** ^**3**^	97 / 88			<0.001
ACD		58 (59.8)	23 (26.1)	
IDA		1 (1.0)	9 (10.2)	
ACD+IDA		10 (10.3)	3 (3.4)	
No anemia		28 (28.9)	53 (60.2)	
**Iron deficiency** ^**4**^				
CRP containing index <0	99 / 89	80 (80.8)	86 (96.6)	0.006
Ferritin <30 μg/L	101 / 89	2 (2.0)	13 (14.6)	0.02
MCV <80 f/L	102 / 98	67 (65.7)	44 (44.9)	0.009
MCH <27.5 g/dL	102 / 98	75 (73.5)	65 (66.3)	0.27
MCHC <32 g/dL	102 / 98	23 (22.6)	30 (30.6)	0.23
sTfR >2.5 mg/L	89 / 75	17 (19.1)	3 (4.0)	0.03
sTfR index >1.5	89 / 75	4 (4.5)	8 (10.7)	0.14
Hepcidin >20 ng/mL	81 / 65	62 (76.5)	23 (35.4)	0.002

ACD, anemia of chronic disease; ACD+IDA, multifactorial anemia (ACD+IDA); IDA, iron deficiency anemia; CRP, C-reactive protein; MCV, mean corpuscular volume; MCH, mean corpuscular hemoglobin; sTfR, soluble transferrin receptor

^1^ Participants with an available laboratory result

^2^ Accounts for case-control matching

^3^ Based on algorithm in S1 Fig

^4^ Based on single laboratory parameters (e.g., independent from hemoglobin levels)

**Fig 1:**
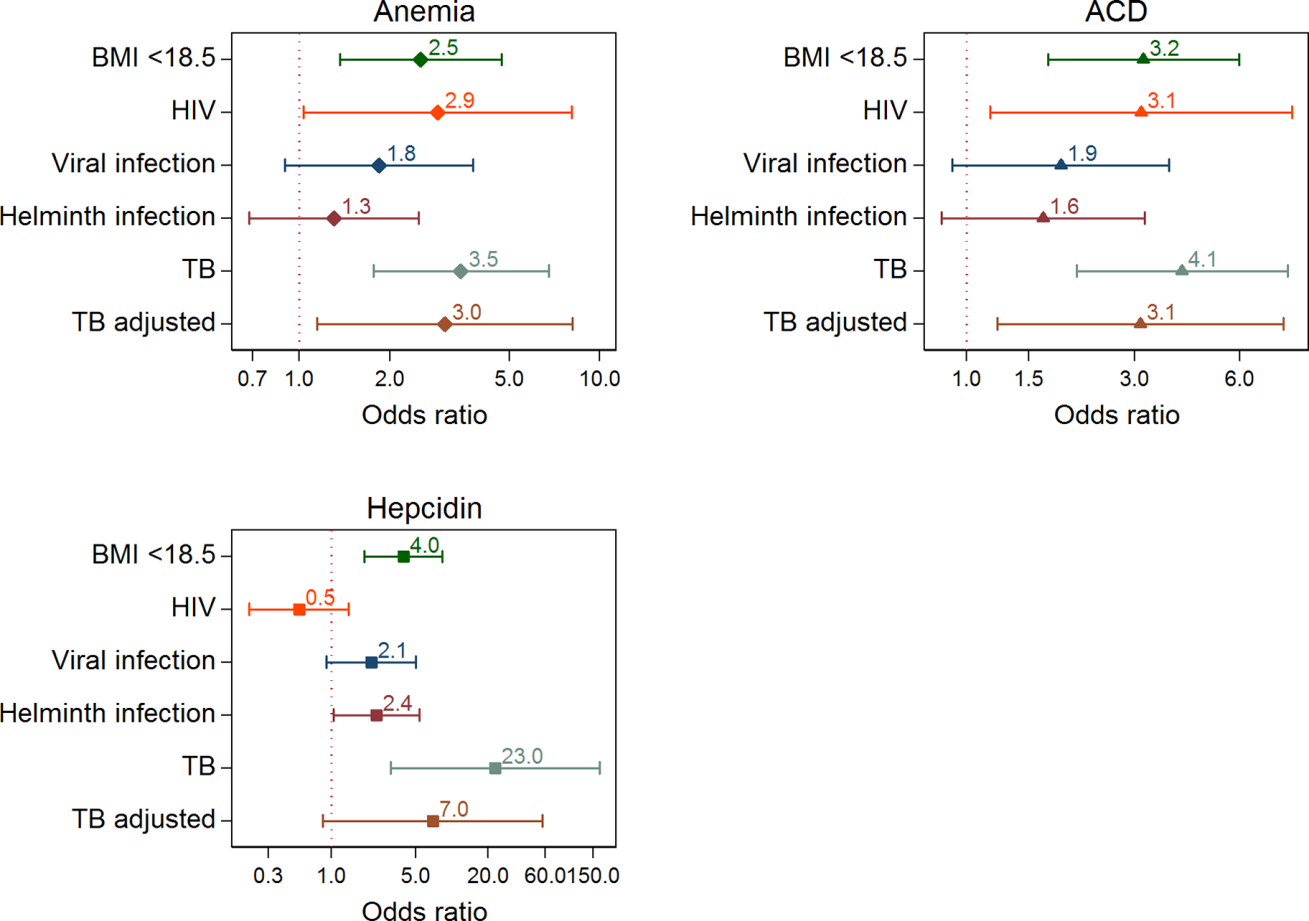
Associations of anemia with patient characteristics and coinfections (HIV, viral respiratory and helminth infection). Anemia based on hemoglobin levels vs. no anemia (“Anemia”); anemia of chronic disease vs. all other forms (“ACD”); and anemia based on hepcidin levels (>20 ng/mL) vs. no anemia (“Hepcidin”). BMI, body mass index; OR, odds ratio; TB, tuberculosis. Estimates from logistic regression models (BMI, HIV, viral and helminth infection) and conditional regression models (TB). TB adjusted for BMI and HIV. ORs on log scale.

Both low BMI (<18.5 mg/kg) and HIV infection, but not viral or helminth coinfections, were risk factors for anemia (defined by hemoglobin levels) and ACD (Fig 1). In multivariate analyses adjusted for BMI and HIV, TB was a risk factor for all types of anemia: aOR 3.1 (95% confidence interval [CI] 1.2–8.1, p = 0.03) for anemia based on hemoglobin levels, aOR 3.1 (95% CI 1.2–8.0, p = 0.02) for ACD, and aOR 7.0 (95% CI 0.9–57.0, p = 0.07) for anemia based on hepcidin levels.

### Associations between hepcidin concentration and coinfections

The overall median hepcidin concentration was 27.3 ng/mL (IQR 9.2–81.4). Hepcidin levels were significantly increased in cases compared to controls (median 63.7 ng/mL, IQR [22–121.9] vs. 14.2 ng/mL, IQR [4.5–27.4], p = 0.003). Hepcidin levels were also higher in men and patients with moderate and severe anemia (S2–S4 Tables). Associations between hepcidin levels and hematological, iron, and inflammatory parameters are shown in S6 Table. Ferritin, transferrin, and the inflammatory markers procalcitonin and CRP were all associated with hepcidin levels. The concentration of sTfR was negatively associated with hepcidin levels in controls, but to a lesser extent in cases.

**Fig 2:**
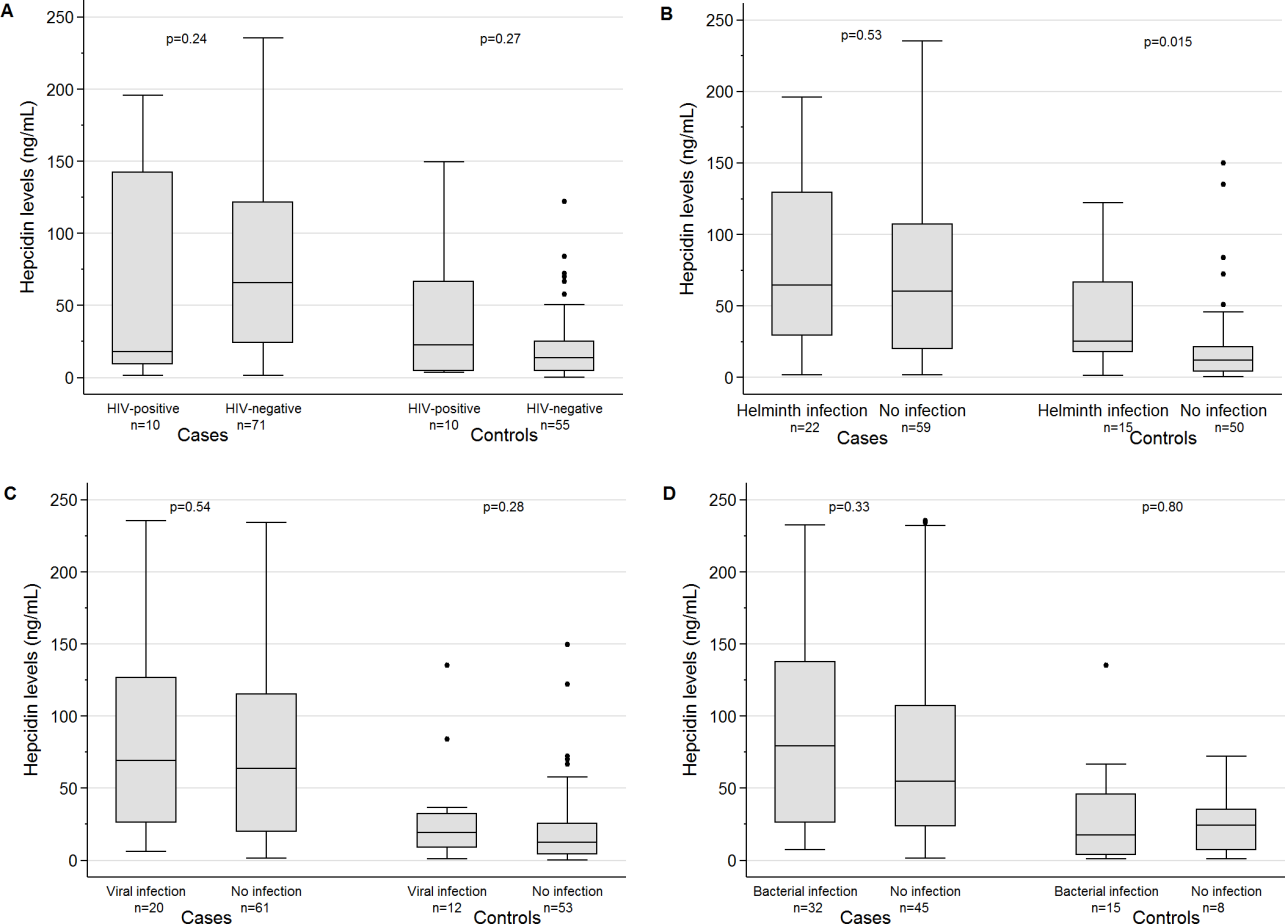
Box plots of hepcidin levels and coinfections in TB patients (cases) and household controls without TB (controls). **A:** Coinfection with HIV; **B:** Coinfection with any helminth infection (soil-transmitted and intestinal helminths). **C:** Coinfection with viral respiratory pathogens. **D:** Coinfection with bacterial respiratory pathogens. P values for comparison between cases and controls: p<0.001. All study participants with available laboratory results were included.

There was no evidence for a cumulative effect of coinfections with HIV, helminths, and respiratory pathogens on hepcidin levels (Fig 2). However, hepcidin levels were increased in controls with helminth infection compared to controls without helminth infection, though this effect was not seen among TB cases (Fig 2B). *Stronglyloides stercoralis* infection was the predominant driver of increased hepcidin levels in controls with helminth infections (S4 Fig).

### Associations between hepcidin concentrations, progression to disease, and disease severity

**Fig 3:**
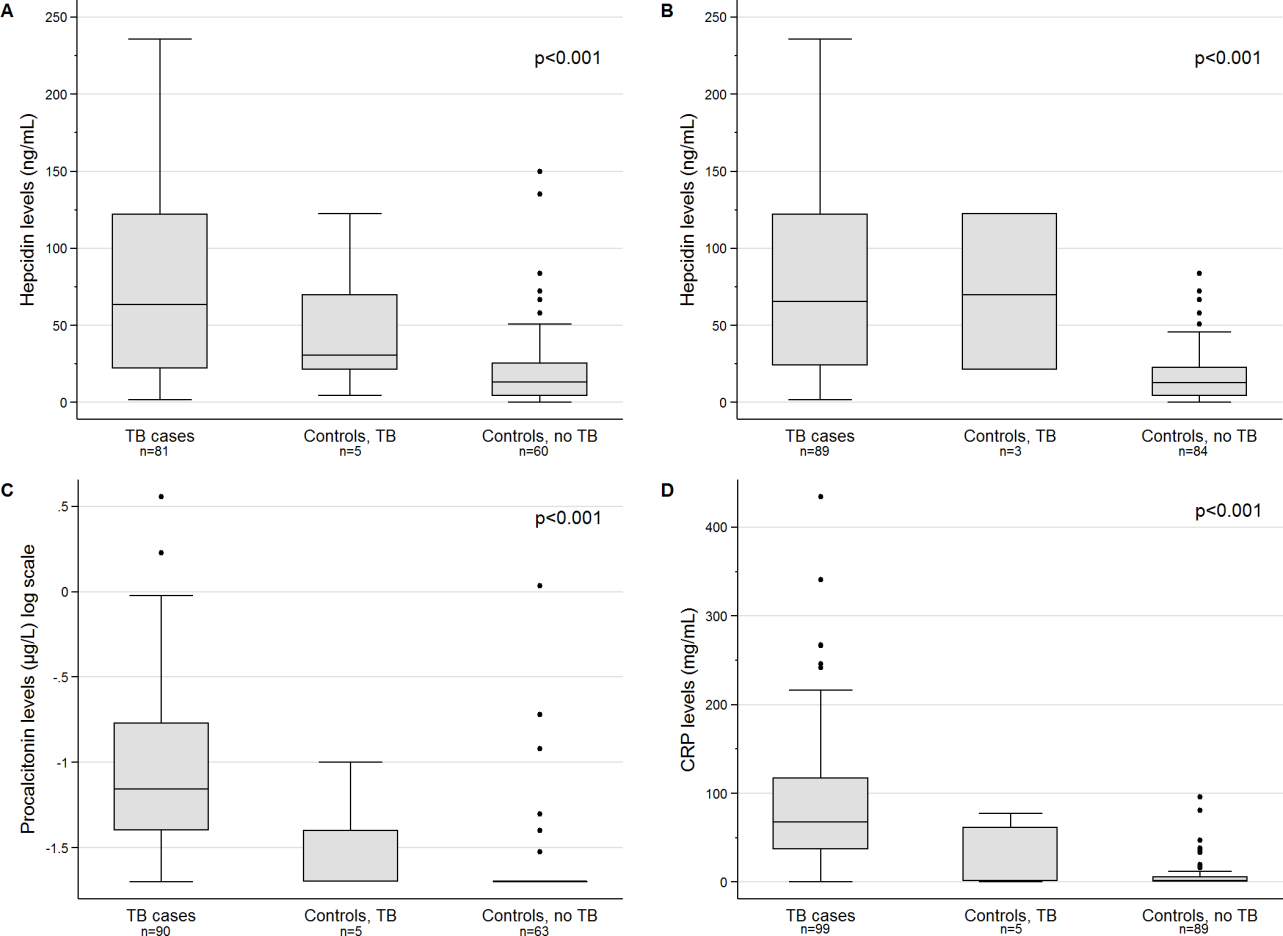
Box plots of hepcidin levels and inflammatory parameters in TB patients (cases), household controls who developed TB within 12 months after enrolment (controls, TB), and household controls who did not develop TB (controls, no TB), at the time of TB diagnosis or enrolment. **A:** Hepcidin concentrations (ng/mL) among all study participants; **B:** Hepcidin concentrations among HIV-negative participants only. **C:** Procalcitonin concentrations (μg/L). **D:** C-reactive protein (CRP) concentrations (mg/L). All study participants with available laboratory results were included: hepcidin (n = 146), procalcitonin (n = 158), CRP (n = 193). *P* values: tests for trend across groups.

Hepcidin levels were significantly higher in TB cases and controls who developed TB during follow-up compared to controls who did not develop TB (test for trend, p<0.001, Fig 3A), even when restricting the comparison to HIV-negative study participants (p<0.001) (Fig 3B). Furthermore, the inflammation markers procalcitonin and CRP were also increased in TB cases and controls who developed TB (Fig 3C and 3D). In explorative analyses, the distribution of hematological, iron and inflammatory parameters differed between cases and controls who developed and did not develop TB within 12 months (S3 and S5 Tables). The association of increased hepcidin levels and progression to active TB was further confirmed by the regression analysis (Table 4), which showed an association between increasing hepcidin levels and TB even when taking HIV infection into account (coefficient 0.65, 95% CI 0.45–0.85, p<0.001). Hepcidin concentrations were also positively associated with clinical severity of TB as defined by the mycobacterial load in the sputum and the TB symptom scoring. Similar associations were found for the inflammatory marker procalcitonin as well as hemoglobin.

**Table 4:** Associations between log hepcidin, procalcitonin, and hemoglobin levels with progression from exposed individuals to TB and TB disease severity among TB cases (cases) and controls who did (controls, TB) or did not develop TB (controls, no TB) during follow-up.

	Hepcidin (ng/mL)		Procalcitonin (μg/L)		Hemoglobin (g/dL)	
	Coefficient (95% CI)	*P* value ^1^	Coefficient (95% CI)	*P* value ^1^	Coefficient (95% CI)	*P* value ^1^
**Progression to TB** ^**2**^ ^**3**^		<0.001		<0.001		<0.001
Controls, no TB	Ref		Ref		Ref	
Controls, TB	0.36 (-0.47–1.18)		0.54 (-0.10–1.18)		-0.02 (-0.10 to 0.06)	
Cases	0.65 (0.45–0.85)		0.54 (0.40–0.69)		-0.04 (-0.06 to -0.02)	
**TB symptoms scoring**		<0.001		<0.001		<0.001
No TB	Ref		Ref		Ref	
Mild TB symptoms	0.47 (0.21–0.72)		0.47 (0.28–0.66)		-0.02 (-0.04 to 0.005)	
Severe TB symptoms	0.84 (0.51–1.17)		0.58 (0.36–0.81)		-0.07 (-0.10 to -0.04)	
**TB bacterial load** ^**4**^		<0.001		<0.001		<0.001
No TB	Ref		Ref		Ref	
Low bacterial load	0.52 (0.22–0.83)		0.48 (0.28–0.69)		-0.04 (-0.06 to -0.008)	
High bacterial load	0.68 (0.40–0.97)		0.55 (0.34–0.77)		-0.04 (-0.07 to -0.02)	

95% CI, 95% confidence interval; Ref, reference group; TB, tuberculosis. Estimations derived from a regression model with hepcidin (log10) as the outcome. The model “TB and progression to TB” includes study participant group (TB cases, controls with TB, controls without TB) and HIV infection.

^1^ Accounts for case-control matching

^2^ Coefficients adjusted for HIV infection

^3^ TB patients (cases), household contact controls (controls), household contact controls who developed TB within 12 months after enrolment (controls, TB), and household controls without TB (controls, no TB).

^4^ Sputum smear microscopy (quantitative scoring): scant and 1+ (low bacterial load), 2+ and 3+ (high bacterial load)

## Discussion

In our matched case-control study of TB cases and household contact controls without TB, we demonstrated that ACD was the predominant cause of anemia among TB cases. This is in line with previous observations that anemia is mainly due to chronic disease [26,29,32], and ACD has been shown to be the predominant cause of anemia in HIV-positive TB patients [33]. This type of anemia is largely due to the underlying chronic inflammatory state existing in chronic diseases.

Diagnosis of ID in chronic inflammatory diseases such as TB is challenging. For example, using ferritin as a single parameter could underestimate ID because ferritin is upregulated in inflammation [34]. Helminths infections, especially *Strongyloides stercoralis*, low dietary iron intake and absorption from monotonous, cereal-based diets contribute to the burden of anemia [5], and may be the reason for the IDA observed in the control group. We did not find a significant association between TB disease and IDA, which is potentially due to tight regulation of iron metabolism by inflammation, existing iron recycling from senescent red blood cells, and absorption of iron by enterocytes [10].

The sTfR concentration was overall negatively associated with hepcidin levels, but to a lesser extent in cases, which suggests that chronic infection with TB interferes in the regulatory mechanisms. This is in agreement with previous reports on the influence of inflammation on sTfR levels [35]. The higher concentrations of sTfR observed in TB cases compared to controls suggest an erythropoetic stimulus and a demand of iron in TB patients. The two different signals, elevated hepcidin levels causing hypoferremia and the erythropoetic stimulus, indicate a delicate balancing act for iron that is required during chronic infection: both deficiency and excess of iron [10] can increase the risk of worse TB treatment outcomes [2].

Hepcidin was associated with TB disease severity and progression to active TB, but not with HIV, helminth, and respiratory pathogen coinfections. Infections with viruses such as hepatitis B and C have been observed to have little effect on hepcidin homeostasis [12]. Yet at the time of recruitment, we observed hepcidin levels that were already elevated well before active disease appeared in controls who developed TB during follow-up. This finding held even when excluding HIV-positive patients and adjusting the regression model to HIV infection as the most important risk factor for progression from infection to active TB [36]. This suggests that altered iron metabolism long precede active disease. Regarding the hormone as a participant in innate immune response, we noted that, other inflammatory markers procalcitonin and CRP were also increased before development of active TB. Elevated levels of all three of these markers suggest that the body is already in an inflammatory state as previously reported [6,12,32,37], but the association of hepcidin with procalcitonin found in our study has not been reported so far.

Anemia resulting from chronic inflammation has a complex pathophysiology depending on the underlying disease process. Previous studies have found results similar to ours [11,37], but have not considered coinfections, inflammation parameters, follow-up of TB-exposed individuals (progression to disease) and clinical presentation, which are novel in our study. Our results are also in line with reports from South Africa, which focused only on HIV-positive TB patients [6,37]. There are concerns of the clinical benefit from general supplementation of iron in anemic TB patients [38]. Moreover, a clinical trial on iron supplementation in TB patients showed a positive effect on hematological indices in the intervention group compared to the placebo group after one month, but these effects disappeared after two and six months [39]. This indicates that with the resolution of infection (and the decrease in hepcidin levels), adequate concentrations of iron become available in the blood of the majority of TB patients by the release of sequestered iron and improved iron absorption. However, a small proportion of TB patients with underlying ID may still benefit from iron supplementation, but the accurate detection of ID in these patients remains an unresolved problem. Low hepcidin levels may help distinguish patients with IDA versus ACD [8], and patients who may benefit most from iron supplementation. More work is needed to understand the clinical utility of hepcidin in the context of coinfections [13].

Our study had some limitations, notably among them no follow-up serum taken after completion of TB treatment to further evaluate the role of hepcidin and other inflammatory markers in TB. However, we were able to collect a wide range of clinical data that encompassed information on coinfections including HIV, helminths, and viral and bacterial respiratory pathogens, and laboratory parameters that included inflammatory and hematological markers. The study design allowed comparison of cases versus controls with sufficient power. We did not assess the β-thalassemia trait, which could have influenced the hematological parameters [40]. Finally, the sample size of household contact controls was relatively small.

In conclusion, hepcidin was marginally upregulated by coinfections other than *M*. *tuberculosis*, and could be a marker identifying more severe TB disease and high-risk individuals among persons exposed to TB. In the management of anemia in TB patients, a small proportion would benefit from iron supplementation [2,3]. However, iron supplementation must be approached cautiously because the functional iron deficit that is observed in TB patients with anemia is mostly temporary due to sequestration of iron in the cells and reversible during TB treatment [39]. Clinical management of anemia and nutritional interventions among TB patients to improve the clinical course and TB treatment outcomes in under-resourced settings need further investigation. Future studies with larger sample sizes and also including additional biomarkers could further explore and confirm our findings.

## Supporting information

S1 Fig Anemia case definitions according to iron deficiency and chronic disease. (DOCX)

S2 Fig WHO anemia classification in cases and controls. (DOCX)

S3 Fig Box plots of hepcidin levels (ng/mL) in TB patients (cases) and household controls without TB (controls), stratified by sex (A) and by WHO anemia severity classification (B).
(DOCX)

S4 Fig Box plots of hepcidin levels (ng/mL) in TB patients (cases) and household controls, stratified by *Strongyloides stercoralis* infection. (DOCX)

S1 Table Detection of respiratory viral and bacterial pathogens using a multiplex real-time PCR in nasopharyngeal swabs. (DOCX)

S2 Table Hematological, iron and inflammatory parameters according to anemia severity among cases and controls. (DOCX)

S3 Table Hematological, iron and inflammatory parameters among cases, controls and controls who developed tuberculosis. (DOCX)

S4 Table Hematological, iron and inflammatory parameters according to sex, among cases and controls. (DOCX)

S5 Table Etiology of anemia and iron deficiency based on single laboratory parameters among TB cases, controls and controls who developed tuberculosis. (DOCX)

S6 Table Associations between log hepcidin levels (ng/mL) and other hematological indices (log scale). (DOCX)
