# Supplementary material for: Anemia in tuberculosis cases and household controls from Tanzania: Contribution of disease, coinfections, and the role of hepcidin
Source: PLoS One. 2018 Apr 20;13(4):e0195985. doi: 10.1371/journal.pone.0195985 (PMC5909902; doi:10.1371/journal.pone.0195985)
Supplement: S1 Fig — (DOCX) [file pone.0195985.s001.docx]

**S1 Fig. Anemia case definitions according to iron deficiency and chronic disease.** Kerkhoff et al. [Ref. 1].

ACD, anemia of chronic disease; Hb, hemoglobin; IDA, iron deficiency anemia; sTfR, soluble transferrin receptor

**Reference**

1. Kerkhoff AD, Meintjes G, Opie J, et al. Anaemia in patients with HIV-associated TB: relative contributions of anaemia of chronic disease and iron deficiency. Int J Tuberc Lung Dis. **2016**; 20(2):193–201.
