# Supplementary material for: Anemia in tuberculosis cases and household controls from Tanzania: Contribution of disease, coinfections, and the role of hepcidin
Source: PLoS One. 2018 Apr 20;13(4):e0195985. doi: 10.1371/journal.pone.0195985 (PMC5909902; doi:10.1371/journal.pone.0195985)
Supplement: S2 Fig — (DOCX) [file pone.0195985.s002.docx]

**S2 Fig. WHO anemia classification in cases and controls.**

Numbers on the bars indicate absolute numbers.

**
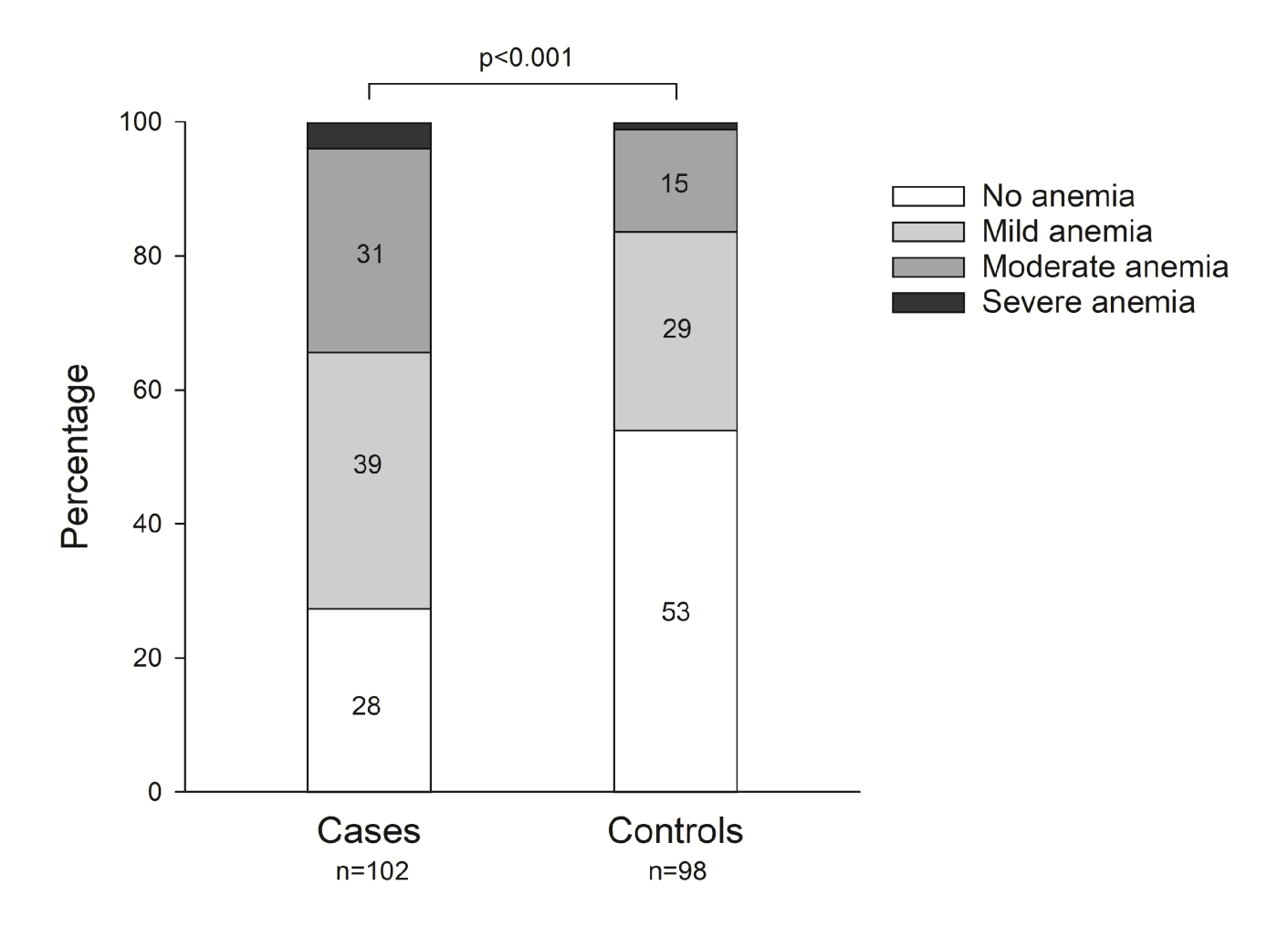
**

*P* value was obtained using a chi-square test across the two groups
