# Supplementary material for: Anemia in tuberculosis cases and household controls from Tanzania: Contribution of disease, coinfections, and the role of hepcidin
Source: PLoS One. 2018 Apr 20;13(4):e0195985. doi: 10.1371/journal.pone.0195985 (PMC5909902; doi:10.1371/journal.pone.0195985)
Supplement: S3 Fig — Box plots of hepcidin levels (ng/mL) in TB patients (cases) and household controls without TB (controls), stratified by sex (A) and by WHO anemia severity classification (B). (DOCX) [file pone.0195985.s003.docx]

**S3 Fig. Box plots of hepcidin levels (ng/mL) in TB patients (cases) and household controls without TB (controls), stratified by sex (A) and by WHO anemia severity classification (B).**


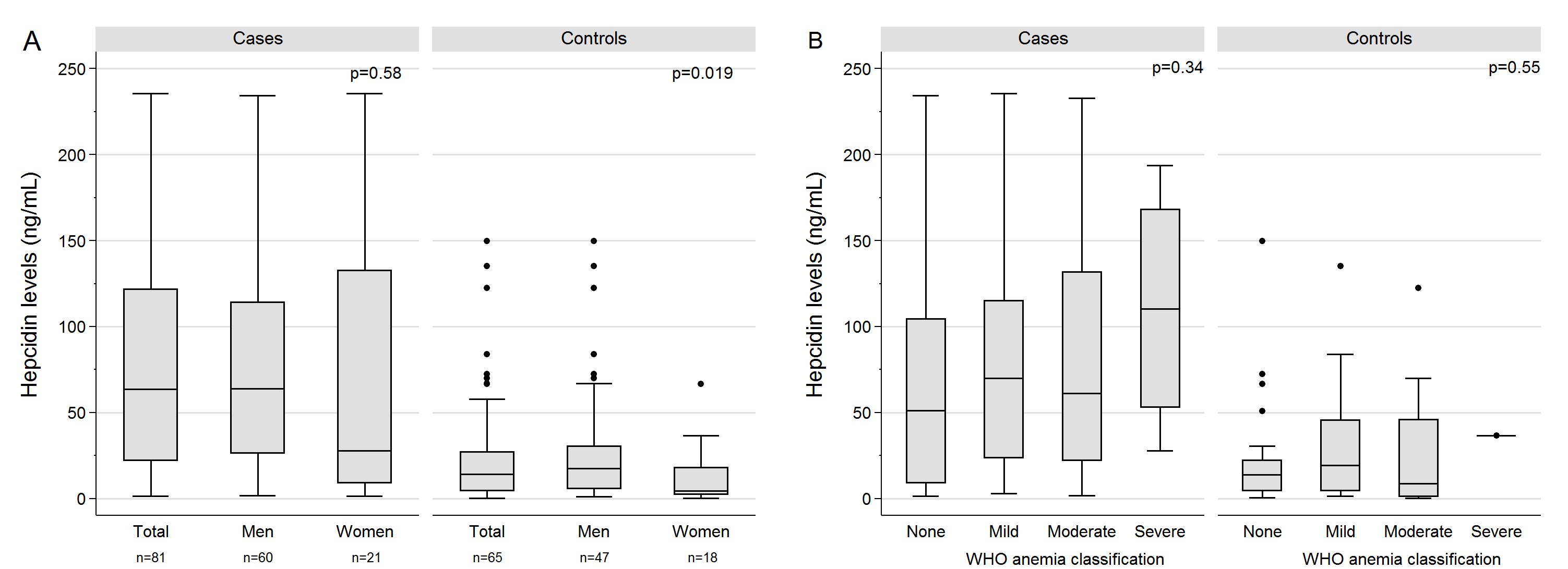


*P* values were obtained using Kruskal-Wallis tests
