# Supplementary material for: Anemia in tuberculosis cases and household controls from Tanzania: Contribution of disease, coinfections, and the role of hepcidin
Source: PLoS One. 2018 Apr 20;13(4):e0195985. doi: 10.1371/journal.pone.0195985 (PMC5909902; doi:10.1371/journal.pone.0195985)
Supplement: S1 Table — (DOCX) [file pone.0195985.s005.docx]

**S1 Table. Detection of respiratory viral and bacterial pathogens using a multiplex real-time PCR in nasopharyngeal swabs.**

| Viral species |  | Bacterial species |
| --- | --- | --- |
| Anyplex II RV16 (panels A and B) |  | Allplex respiratory panel 4 |
| Adenovirus  Influenza A  Influenza B  Rhinovirus A/B/C  Respiratory syncytial virus A  Respiratory syncytial virus B  Parainfluenza virus 1  Parainfluenza virus 2  Parainfluenza virus 3  Parainfluenza virus 4  Bocavirus 1/2/3/4  Metapneumovirus  Coronavirus 229  Coronavirus OC4  Coronavirus NL63  Enterovirus |  | *Mycoplasma pneumonia*  *Chlamydophila pneumoniae*  *Legionella pneumophila*  *Haemophilus influenzae*  *Streptococcus pneumoniae*  *Bordetella pertussis*  *Bordetella parapertussis* |
