# Supplementary material for: Anemia in tuberculosis cases and household controls from Tanzania: Contribution of disease, coinfections, and the role of hepcidin
Source: PLoS One. 2018 Apr 20;13(4):e0195985. doi: 10.1371/journal.pone.0195985 (PMC5909902; doi:10.1371/journal.pone.0195985)
Supplement: S2 Table — (DOCX) [file pone.0195985.s006.docx]

**S2 Table. Hematological, iron and inflammatory parameters according to anemia severity among cases and controls.**

| **Parameter** |  | **Cases, median (IQR)** | | | |  | **Controls, median (IQR)** | | | |
| --- | --- | --- | --- | --- | --- | --- | --- | --- | --- | --- |
|  |  | **None**  (n=28) | **Mild**  (n=39) | **Mod/severe**  (n=35) | ***P* value** |  | **None**  (n=53) | **Mild**  (n=29) | **Mod/severe**  (n=16) | ***P* value** |
| Iron (µmol/L) |  | 6.7 (3.7-12.8) | 4.5 (3.6-6.1) | 3.8 (3.0-6.1) | 0.02 |  | 15.3 (10.8-19.1) | 11.6 (8.9-14.3) | 8.6 (5.5-13.9) | 0.003 |
| Ferritin (ng/mL) |  | 222.2 (128.1-566.5) | 384.5 (199.1-790.1) | 333.7 (181.0-524.0) | 0.14 |  | 112.1 (68.0-152.5) | 88.8 (44.2-169.6) | 70.9 (14.9-171.0) | 0.47 |
| Soluble transferrin receptor (mg/L) |  | 1.4 (1.4-1.7) | 1.9 (1.5-2.1) | 2.1 (1.6-2.9) | 0.006 |  | 1.4 (1.2-1.6) | 1.4 (1.2-1.8) | 1.9 (1.5-2.3) | 0.044 |
| Transferrin (g/L) |  | 1.8 (1.5-2.2) | 1.7 (1.4-1.9) | 1.4 (1.3-1.8) | 0.009 |  | 2.6 (2.4-2.8) | 2.4 (2.0-2.7) | 2.3 (2.1-2.) | 0.1 |
| Hepcidin (ng/mL) |  | 51.0 (9.2-104.5) | 69.9 (23.6-115.2) | 70.1 (23.1-132.7) | 0.53 |  | 13.9 (4.6-22.3) | 19.5 (4.5-45.8) | 12.7 (1.1-36.6) | 0.59 |
| CRP (mg/L) |  | 54.4 (19.8-115.5) | 80.5 (30-112.3) | 89.2 (49.9-145.2) | 0.16 |  | 0.9 (0.6-3.3) | 3.3 (0.7-10.2) | 4.1 (0.6-19.3) | 0.024 |
| Procalcitonin (µg/L) |  | 0.05 (0.02-0.21) | 0.07 (0.04-0.2) | 0.08 (0.05-0.19) | 0.31 |  | 0.020 (0.02-0.02) | 0.02 (0.02-0.04) | 0.02 (0.02-0.05) | 0.25 |
| Hemoglobin (g/dL) |  | 13.7 (13.3-14.5) | 12.3 (11.5-12.7) | 9.7 (8.5-10.3) | 0.001 |  | 14.1 (13.5-15.0) | 12.1 (11.5-12.6) | 9.5 (8.6-10.3) | 0.001 |
| MCV (f/L) |  | 78.4 (71.4-83.5) | 78.6 (71.6-84.3) | 70.5 (63.4-76.9) | 0.006 |  | 83.9 (78.7-87.2) | 78.0 (72.3-83.5) | 78.0 (65.7-85.6) | 0.006 |
| MCH (pg/cell) |  | 26.4 (24.1-28.4) | 26.1 (23.4-27.7) | 22.4 (20.5-24.5) | <0.001 |  | 27.8 (25.8-29.8) | 24.2 (23.0-26.8) | 22.9 (20.5-26.1) | <0.001 |
| MCHC (g/dL) |  | 34.0 (33.6-34.8) | 33.1 (32.3-34.2) | 32.2 (31.4-32.9) | <0.001 |  | 33.4 (32.7-34.8) | 32.2 (31.0-32.9) | 31.1 (29.7-32.3) | <0.001 |
| Red blood cell distribution width (f/L) |  | 14.6 (13.1-16.2) | 14.6 (13.6-15.9) | 16.7 (14.9-19.4) | 0.005 |  | 14.1 (13.1-14.9) | 14.7 (13.9-16.3) | 15.6 (14.7-19.3) | 0.008 |

CRP, C-reactive protein; MCV, mean corpuscular volume; MCH, mean corpuscular hemoglobin; MCHC, mean corpuscular hemoglobin concentration; Mod/severe, moderate and severe anemia (WHO classification)

*P* values were obtained using Kruskal-Wallis tests
