# Supplementary material for: Anemia in tuberculosis cases and household controls from Tanzania: Contribution of disease, coinfections, and the role of hepcidin
Source: PLoS One. 2018 Apr 20;13(4):e0195985. doi: 10.1371/journal.pone.0195985 (PMC5909902; doi:10.1371/journal.pone.0195985)
Supplement: S3 Table — (DOCX) [file pone.0195985.s007.docx]

**S3 Table. Hematological, iron and inflammatory parameters among cases, controls and controls who developed tuberculosis** (also see Table 2)**.**

| **Parameter** | **No. included ^1^** | **Median (IQR)** | | |
| --- | --- | --- | --- | --- |
| (unit) | Cases / Controls, TB ^2^ / Controls | Cases | Controls, TB ^2^ | Controls |
| Iron (µmol/L) | 99 / 5 / 86 | 4.4 (3.3-7.1) | 11.6 (4.0-11.7) | 13 (9.0-17.7) |
| Ferritin (ng/mL) | 101 / 5 / 84 | 309.8 (162.2-601.2) | 171 (107.4-263.6) | 100 (59.1-144) |
| sTfR (mg/L) | 89 / 5 / 70 | 1.8 (1.4-2.2) | 1.5 (1.4-1.9) | 1.4 (1.2-1.8) |
| Transferrin (g/L) | 99 / 5 / 89 | 1.6 (1.4-2.0) | 2.3 (1.8-2.3) | 2.5 (2.3-2.8) |
| Hepcidin (ng/mL) | 81 / 5 / 60 | 63.7 (22.0-121.9) | 30.5 (21.1-69.9) | 13.2 (4.2-25.5) |
| CRP (mg/L) | 99 / 5 / 89 | 67.8 (36.5-116.9) | 1.6 (0.9-61.2) | 1.5 (0.6-5.8) |
| Procalcitonin (µg/L) | 90 / 5 / 63 | 0.07 (0.04-0.17) | 0.04 (0.02-0.04) | 0.019 (0.019-0.02) |
| Hemoglobin (g/dL) | 102 / 5 / 93 | 12.1 (10.3-12.9) | 11.1 (10.6-11.3) | 13.1 (11.8-14.3) |
| MCV (f/L) | 102 / 5 / 93 | 75.5 (68.6-82.8) | 85.3 (77.0-94.0) | 81 (76.0-86.0) |
| MCH (pg/cell) | 102 / 5 / 93 | 25.0 (22.4-27.5) | 28.1 (25.5-28.7) | 26.3 (23.8-29.1) |
| MCHC (g/dL) | 102 / 5 / 93 | 33.1 (32.0-34.0) | 32.9 (32.6-33.0) | 32.7 (31.2-33.9) |
| Red blood cell distribution width (f/L) | 63 / 5 / 77 | 14.9 (13.8-16.7) | 14.6 (14.1-15.8) | 14.7 (13.5-15.6) |

CRP, C-reactive protein; MCV, mean corpuscular volume; MCH, mean corpuscular hemoglobin; MCHC, mean corpuscular hemoglobin concentration; sTfR, soluble transferrin receptor

^1^ Patients with an available laboratory result

^2^ Controls who developed tuberculosis
