# Supplementary material for: Anemia in tuberculosis cases and household controls from Tanzania: Contribution of disease, coinfections, and the role of hepcidin
Source: PLoS One. 2018 Apr 20;13(4):e0195985. doi: 10.1371/journal.pone.0195985 (PMC5909902; doi:10.1371/journal.pone.0195985)
Supplement: S4 Table — (DOCX) [file pone.0195985.s008.docx]

**S4 Table. Hematological, iron and inflammatory parameters according to sex, among cases and controls.**

| **Parameter** |  | **Cases**  **median (IQR)** | | |  | **Controls**  **median (IQR)** | | |
| --- | --- | --- | --- | --- | --- | --- | --- | --- |
|  |  | **Male**  (n=78) | **Female**  (n=24) | ***P* value** |  | **Male**  (n=74) | **Female**  (n=24) | ***P* value** |
| Iron (µmol/L) |  | 4.4 | 4.0 (3.0-9.2) | 0.9 |  | 14.3 (9.4-19.0) | 9.8 (6.3-13.1) | 0.008 |
| Ferritin (ng/mL) |  | 424.5 (212.3-68.2) | 160.9 (117.6-309.8) | 0.001 |  | 120.3 (76.2-180.8) | 32.7 (14.9-72.8) | <0.001 |
| Soluble transferrin receptor (mg/L) |  | 1.7 (1.4-2.1) | 1.9 (1.6-2.8) | 0.1 |  | 1.3 (1.1-1.6) | 1.9 (1.4-2.2) | <0.001 |
| Transferrin (g/L) |  | 1.6 (1.4-1.9) | 1.6 (1.3-2.2) | 0.9 |  | 2.4 (2.3-2.7) | 2.7 (2.3-3.3) | 0.015 |
| Hepcidin (ng/mL) |  | 64.0 (26.4-114.4) | 27.8 (9.2-132.7) | 0.58 |  | 17.5 (5.6-30.5) | 4.6 (2.4-18.3) | 0.019 |
| CRP (mg/L) |  | 76.3 (41.3-115.1) | 58.8 (29.2-158) | 0.7 |  | 1.5 (0.6-7.2) | 1.7 (0.6-5.5) | 0.9 |
| Procalcitonin (µg/L) |  | 0.09 (0.04-0.19) | 0.06 (0.03-0.09) | 0.3 |  | 0.02 (0.02-0.03) | 0.02 (0.02-0.03) | 0.7 |
| Hemoglobin (g/dL) |  | 12.4 (10.8-13.3) | 10.1 (8.4-11.6) | <0.001 |  | 13.6 (12.5-14.7) | 1.2 (10.1-12.1) | <0.001 |
| MCV (f/L) |  | 77.2 (686-83.4) | 73.4 (65.5-79.5) | 0.1 |  | 83.5 (77.2-87.0) | 75.5 (67.5-81.6) | 0.001 |
| MCH (pg/cell) |  | 25.7 (22.8-27.7) | 23.5 (21.3-25.9) | 0.03 |  | 26.9 (24.2-29.7) | 23.9 (20.9-26.2) | <0.001 |
| MCHC (g/dL) |  | 33.3 (32.4-34.1) | 31.8 (30.9-33.3) | 0.004 |  | 33.0 (31.7-34.2) | 32.2 (29.9-33.0) | 0.01 |
| Red blood cell distribution width (f/L) |  | 14.6 (13.6-16.2) | 16.8 (14.8-19.8) | 0.004 |  | 14.5 (13.3-15.6) | 14.8 (13.9-16.7) | 0.1 |

CRP, C-reactive protein; IQR, interquartile range; MCV, mean corpuscular volume; MCH, mean corpuscular hemoglobin;

MCHC, mean corpuscular hemoglobin concentration

*P* values were obtained using Kruskal-Wallis tests
