# Supplementary material for: Anemia in tuberculosis cases and household controls from Tanzania: Contribution of disease, coinfections, and the role of hepcidin
Source: PLoS One. 2018 Apr 20;13(4):e0195985. doi: 10.1371/journal.pone.0195985 (PMC5909902; doi:10.1371/journal.pone.0195985)
Supplement: S5 Table — (DOCX) [file pone.0195985.s009.docx]

**S5 Table. Etiology of anemia and iron deficiency based on single laboratory parameters among TB cases, controls and controls who developed tuberculosis** (also see Table 3).

| **Classification** | **No. included ^1^** | **n (%)** | | |
| --- | --- | --- | --- | --- |
|  | Cases / Controls TB ^2^ / Controls | Cases | Controls, TB ^2^ | Controls |
| **Cause-specific anemia ^3^** | 97 / 5 / 83 |  |  |  |
| ACD |  | 58 (59.8) | 3 (60) | 20 (24.1) |
| IDA |  | 1 (1.0) | 0 | 9 (10.8) |
| ACD+IDA |  | 10 (10.3) | 1 (20.0) | 2 (2.4) |
| No anemia |  | 28 (28.9) | 1 (20.0) | 52 (62.7) |
| **Iron deficiency ^4^** |  |  |  |  |
| CRP containing index <0 | 99 / 5 / 84 | 80 (80.8) | 4 (80.0) | 82 (97.6) |
| Ferritin <30 µg/L | 101 / 5 / 84 | 2 (2.0) | 0 | 13 (15.5) |
| MCV <80 f/L | 102 / 5 / 93 | 67 (65.7) | 2 (40.0) | 42 (45.2) |
| MCH <27.5 g/dL | 102 / 5 / 93 | 75 (73.5) | 2 (40.0) | 63 (67.7) |
| MCHC <32 g/dL | 102 / 5 / 93 | 23 (22.6) | 1 (20.0) | 29 (31.2) |
| sTfR >2.5 mg/L | 89 / 5 / 70 | 17 (19.1) | 0 | 3 (4.3) |
| sTfR index >1.5 | 89 / 5 / 70 | 4 (4.5) | 0 | 8 (11.4) |
| Hepcidin >20 ng/mL | 81 / 5 / 60 | 62 (76.5) | 4 (80.0) | 19 (31.7) |

ACD, anemia of chronic disease; ACD+IDA, multifactorial anemia (ACD+IDA); IDA, iron deficiency anemia; CRP, C-reactive protein; MCV, mean corpuscular volume; MCH, mean corpuscular hemoglobin; sTfR, soluble transferrin receptor

^1^ Patients with an available laboratory result

^2^ Controls who developed tuberculosis

^3^ Based on algorithm in S1 Fig.

^4^ Based on single laboratory parameters (e.g., independent from hemoglobin levels)
