# Supplementary material for: Anemia in tuberculosis cases and household controls from Tanzania: Contribution of disease, coinfections, and the role of hepcidin
Source: PLoS One. 2018 Apr 20;13(4):e0195985. doi: 10.1371/journal.pone.0195985 (PMC5909902; doi:10.1371/journal.pone.0195985)
Supplement: S6 Table — (DOCX) [file pone.0195985.s010.docx]

**S6 Table. Associations between log hepcidin levels (ng/mL) and other hematological indices (log scale).**

| **Parameter** | **All** | |  | **Cases** | |  | **Controls** | |
| --- | --- | --- | --- | --- | --- | --- | --- | --- |
|  | Coefficient (95% CI) | *P* value |  | Coefficient (95% CI) | *P* value |  | Coefficient (95% CI) | *P* value |
| Iron (µmol/L) | -1.08 (-1.36 to -0.79) | <0.001 |  | -1.32 (-1.60 to -1.04) | <0.001 |  | 0.33 (-0.31-0.98) | 0.30 |
| Ferritin (ng/mL) | 0.96 (0.83-1.09) | <0.001 |  | 0.75 (0.51-1.00) | <0.001 |  | 0.98 (0.82-1.14) | <0.001 |
| Soluble transferrin receptor (mg/L) | -0.05 (-0.068-0.57) | 0.87 |  | -0.04 (-0.060-0.69) | 0.89 |  | -1.62 (-2.69 to -0.55) | <0.004 |
| Transferrin (g/L) | -2.75 (-3.33 to -2.18) | <0.001 |  | -1.91 (-2.73 to -1.10) | <0.001 |  | -3.92 (-5.46 to -2.37) | <0.001 |
| CRP (mg/L) | 0.54 (0.46-0.62) | <0.001 |  | 0.66 (0.51-0.80) | <0.001 |  | 0.52 (0.33-0.71) | <0.001 |
| Procalcitonin (µg/L) | 0.77 (0.60-0.95) | <0.001 |  | 0.58 (0.37-0.78) | <0.001 |  | 0.89 (0.42-1.36) | <0.001 |
| Hemoglobin (g/dL) | -0.75 (-2.00-0.51) | 0.24 |  | -0.35 (-1.81-1.11) | 0.64 |  | 0.86 (-1.03-2.75) | 0.37 |
| MCV (f/L) | -0.64 (-2.66-1.37) | 0.53 |  | 0.10 (-2.28-2.47) | 0.93 |  | 1.47 (-1.43-4.38) | 0.32 |
| MCH (pg/cell) | 0.21 (-1.53-1.95) | 0.81 |  | -0.06 (-2.23-2.10) | 0.95 |  | 1.97 (-0.29-4.23) | 0.087 |
| MCHC (g/dL) | 3.80 (-0.32-7.93) | 0.071 |  | -1.58 (-7.95-4.78) | 0.62 |  | 4.34 (-0.035-9.02) | 0.069 |

95% CI, 95% confidence interval; CRP, C-reactive protein; MCV, mean corpuscular volume; MCH, mean corpuscular hemoglobin; MCHC, mean corpuscular hemoglobin concentration; sTfR, soluble transferrin receptor

Estimations derived from regression models (on log scale)
